# Investigation of In Vitro Cytocompatibility of Zinc-Containing Coatings Developed on Medical Magnesium Alloys

**DOI:** 10.3390/ma17010209

**Published:** 2023-12-30

**Authors:** Yun Wang, Yuzhi Liu, Yuanyuan Zhu, Fanglei Yu, Rongfang Zhao, Xinying Lai, Haijun Jiang, Tianhong Xu, Ying Zhao, Rongfa Zhang

**Affiliations:** 1 School of Materials and Energy, Jiangxi Science and Technology Normal University, Nanchang 330013, China; 17839410350@163.com (Y.W.); yuanyuanzhu_01@163.com (Y.Z.); zhaorfamy@126.com (R.Z.); l2663360293@163.com (X.L.); jhj20020621@163.com (H.J.); 15079431924@163.com (T.X.); 2 Shenzhen Institute of Advanced Technology, Chinese Academy of Sciences, Shenzhen 518055, China; yz.liu1@siat.ac.cn; 3 R & D Department, Zhejiang Ruigu Biotechnology Co., Ltd., Hangzhou 311121, China; 4 Zhejiang Canwell Medical Co., Ltd., Jinhua 321000, China; fanglei.yu@canwell.com.cn

**Keywords:** magnesium alloys, MAO treatment, zinc-containing coatings, in vitro cytocompatibility, degradation resistance

## Abstract

In a neutral solution, we investigated the effects of Na_2_[ZnEDTA] concentrations at 0, 6, 12, 18, and 24 g/L on surface morphology, chemical composition, degradation resistance, and in vitro cytocompatibility of micro-arc oxidation (MAO) coatings developed on WE43 (Mg-Y-Nd-Zr) magnesium alloys. The results show that the enhanced Na_2_[ZnEDTA] concentration increased the Zn amount but slightly decreased the degradation resistance of MAO-treated coatings. Among the zinc-containing MAO samples, the fabricated sample in the base solution added 6 g/L Na_2_[ZnEDTA] exhibits the smallest corrosion current density (6.84 × 10^−7^ A·cm^−2^), while the sample developed in the solution added 24 g/L Na_2_[ZnEDTA] and contains the highest Zn content (3.64 wt.%) but exhibits the largest corrosion current density (1.39 × 10^−6^ A·cm^−2^). Compared to untreated WE43 magnesium alloys, zinc-containing MAO samples promote initial cell adhesion and spreading and reveal enhanced cell viability. Coating degradation resistance plays a more important role in osseogenic ability than Zn content. Among the untreated WE43 magnesium alloys and the treated MAO samples, the sample developed in the base solution with 6 g/L Na_2_[ZnEDTA] reveals the highest ALP expression at 14 d. Our results indicate that the MAO samples formed in the solution with Na_2_[ZnEDTA] promoted degradation resistance and osseogenesis differentiation of the WE43 magnesium alloys, suggesting potential clinic applications.

## 1. Introduction

Because of a similar density and Young’s modulus to human bone, magnesium alloys can effectively alleviate the stress shielding effects when they are used as medical materials [1,2]. Magnesium alloys can be used to avoid secondary surgery and, therefore, are considered a revolutionary degradable metal for tissue respiration. However, a degradation rate that is too fast in the body restricts their clinical applications. Therefore, it is very meaningful to select appropriate surface techniques to fabricate functionalized coatings on magnesium alloys.

Micro-arc oxidation (MAO) is a widely used surface treatment technique to modify magnesium alloys [3,4,5,6], aluminum alloys [7,8,9,10,11,12], titanium alloys [13,14,15,16], and tantalum alloys [17] by modulating electrolyte compositions and concentrations [18,19,20]. MAO technology can improve the corrosion resistance of magnesium alloys and introduce macro or trace elements such as phosphorus (P) and zinc (Zn) into MAO coating to produce functionalized coating. In addition, MAO treatment can improve the antibacterial ability of magnesium alloys by introducing Ag [21]. In addition to Ag, Zn exhibits good antibacterial ability [22]. Moreover, Zn can promote the growth and development of the human body [23]. Thus, zinc-containing MAO coatings have been fabricated on titanium alloys to enhance biocompatibility and antibacterial performance [24,25,26]. Recently, Liu et al. [24] originally investigated the in vitro long-term antibacterial performance of zinc-containing MAO coatings fabricated on titanium alloys, and the results show that the ROS level of MAO samples was significantly higher untreated Ti6Al4V alloys, even after 14 d of immersion, suggesting great application potential in clinical orthopedics.

Compared to titanium alloys, it is difficult to prepare zinc-containing MAO coatings by one-step MAO treatment on magnesium alloys, and the related reports in this field are scarce [27,28,29,30,31]. The influences of Zn-containing coatings fabricated by one-step MAO treatment on the osseogenic ability and degradation resistance of medical magnesium alloys are rarely reported. As one kind of zinc-containing substance, ethylenediaminetetraacetic acid disodium zinc salt (Na_2_[(OOCCH_2_)_2_NCH_2_CH_2_N(CH_2_COO)_2_Zn], abbreviated Na_2_[ZnEDTA]), exhibits good solubility [32]. In this study, Na_2_[ZnEDTA] was selected as the zinc-containing electrolyte, and MAO coatings were prepared in near-neutral solutions containing 360 g/L hexamethylenetetramine (HMTA). The influences of 0, 6, 12, 18, and 24 g/L Na_2_[ZnEDTA] on coating morphology, chemical compositions, degradation resistance, and in vitro cytocompatibility were systematically studied.

## 2. Experimental

### 2.1. Preparation of MAO Samples

Extruded WE43 (Mg-Y-Nd-Zr) magnesium alloys, whose compositions were reported by our previous manuscript [33], were purchased from Suzhou Chuan Mao Metal Materials Co., Ltd. (Suzhou, China) and used as the substrate. After ground, cleaned, and dried, WE43 samples were MAO-treated using an MAO5D power supply (Chengdu Tongchuang New Materials Surface Engineering and Technology Center, Chengdu, China) using constant current mode. The applied pulse frequency, duty cycle, current density, and treating time were separately 2000 Hz, 35%, 60 mA/cm^2^, and 3 min. In a base solution composed of 6 g/L ammonium bifluoride (NH_4_HF_2_), 25 g/L phosphoric acid (H_3_PO_4_), 12 g/L phytic acid, and 360 g/L HMTA, 0, 6, 12, 18, and 24 g/L Na_2_[ZnEDTA] were separately, and the developed MAO samples with different Zn contents were designated as Zn-0 g/L, Zn-6 g/L, Zn-12 g/L, Zn-18 g/L, and Zn-24 g/L, respectively. Measured by a pH meter (PHS-3C, Shanghai Yoke Instrument Co., Ltd., Shanghai, China), solution pH values of Zn-0 g/L, Zn-6 g/L, Zn-12 g/L, Zn-18 g/L, and Zn-24 g/L were 6.00, 6.10, 6.13, 6.16, and 6.17, respectively.

### 2.2. Surface Characterizations

The morphology and composition of fabricated MAO coatings were measured by a scanning electron microscope (SEM, Zeiss Sigma, Oberkochen, Germany) equipped with an energy-dispersive X-sight spectrophotometer (EDS, Oxford INCA, Oxford, UK). The phase structure of the MAO coatings was measured via X-ray diffraction (Shimadzu XRD-6100, Kyoto, Japan) with Cu Kα radiation in a scanning range from 10° to 80° at a speed of 4°/min.

Potentiodynamic polarization curves were applied to evaluate the degradation resistance of untreated WE43 magnesium alloys and MAO samples using an electrochemical workstation (Reference 600+, Gamry Instruments, Lafayette, LA, USA) at 37 °C in Hanks’ solution at a scanning rate of 1 mV/s. A three-electrode system was adopted with a measured sample (1 cm^2^) as the working electrode, while a saturated calomel electrode (SCE) and a platinum electrode were separately used as the reference electrode and the counter electrode. Potentiodynamic polarization curves were measured from −0.25 to 0.5 V with respect to the open circuit potential (OCP), and the electrochemical parameters were derived by the Tafel extrapolation method [34]. The final results were obtained by measuring five parallel samples to ensure result repeatability.

### 2.3. Cell Culture

The pre-osteoblasts (MC3T3-E1 cells, ATCC, CRL-2592, Manassas, Va, USA) were applied to evaluate the cytocompatibility of untreated WE43 magnesium alloys and MAO coatings. The cells were cultured in a humidified atmosphere of 5% CO_2_ at 37 °C using an alpha-minimum essential medium (α-MEM, Gibco, Waltham, MA, USA) added with 10% fetal bovine serum (FBS, Gibco, Grand Island, NY, USA) and a 1% antimicrobial of penicillin/streptomycin mixture.

### 2.4. Cell Adhesion

Before cell seeding, all measured samples were sealed with silicone, except the surface, with an area of 1 cm × 1 cm, and then washed with acetone and ethanol. After that, the samples were sterilized at 121 °C for 2 h in 75% ethanol for 10 min, and then washed with phosphate-buffered saline (PBS) and put into 24 well plates. The MC3T3-E1 cells at the density of 5.0 × 10^4^/well were seeded on various sample surfaces. After cultivation for 5 and 24 h, respectively, the cells were fixed with a 4% paraformaldehyde solution, permeabilized with 0.1% (*v*/*v*) Triton X-100, and then stained with FITC-Phalloidin and DAPI, respectively. Subsequently, the cytoskeletal actin and cell nuclei were observed by fluorescence microscopy (CX31, Olympus, Tokyo, Japan).

### 2.5. CCK Assay

Prior to the CCK assay, extracts were prepared using soaking samples in an α-MEM serum-free medium (containing 1% antimicrobial of penicillin/streptomycin mixture) with a surface area and medium ratio of 1 mL/cm^2^ in a humidified atmosphere with 5% CO_2_ at 37 °C for 72 h. After being collected and filtered with a 0.22 μm filter membrane, the extracts were then supplemented with 10% fetal bovine serum (FBS) (Gibco). The cytotoxicity of the MAO samples against MC3T3-E1 cells was then quantitatively evaluated using a CCK-8 (Cell Counting Kit, Beyotime, Haimen, China) assay. A total of 100 μL of MC3T3-E1 cell suspension was incubated in 96-well culture plates at 2 × 10^3^/well for 24 h to allow attachment, and then the medium was replaced with 100 μL extract. After being cultured for 3 d, the samples were cleaned twice with PBS. Afterward, 10 μL of CCK-8 was added into each well at 37 °C. After 4 h, the optical density (OD) was detected by a microplate spectrophotometer (Thermo Fisher Scientific, Waltham, MA, USA) at 450 nm.

### 2.6. Alkaline Phosphatase (ALP) Activity

Alkaline phosphatase activity was measured in order to evaluate osteogenic differentiation. MC3T3 cells at a concentration of 3 × 10^4^/well were seeded on the samples in 24-well plates to allow attachment. After 24 h, the media were replaced by fresh culture media containing osteogenic induction fluid. After 7 and 14 d of culture, an Alkaline Phosphatase Assay Kit (Beyotime, Haimen, China) was used to determine the alkaline phosphatase activity of the cells on materials surfaces.

## 3. Results

### 3.1. Surface Characterizations of MAO Coatings

#### 3.1.1. Morphology and Composition of MAO Coatings

**Figure 1:**
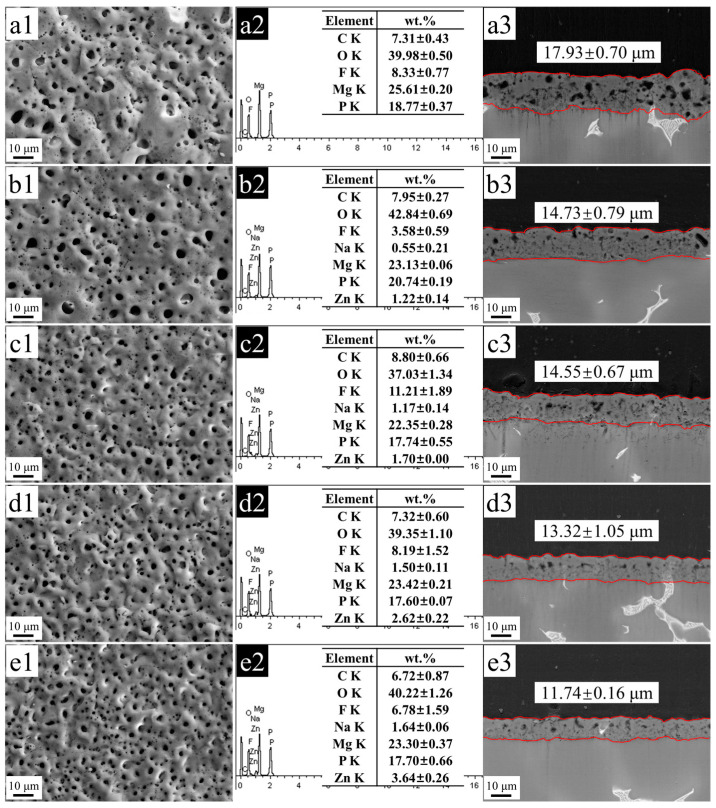
Surface morphologies (**a1**–**e1**), EDS spectra (**a2**–**e2**) and cross-sectional morphology (**a3**–**e3**) of MAO coatings fabricated in the solution containing different concentrations of Na_2_[ZnEDTA]: (**a**) 0 g/L; (**b**) 6 g/L; (**c**) 12 g/L; (**d**) 18 g/L; and (**e**) 24 g/L.

Surface morphologies (Figure 1(a1–e1)), EDS spectra (Figure 1(a2–e2)), and cross-sectional morphologies (Figure 1(a3–e3)) of MAO coatings fabricated in the base solution added different Na_2_[ZnEDTA] concentrations, which are shown in Figure 1. According to Figure 1(a1), Zn-0 g/L was the roughest with uneven micropores, and the largest pore size was in the range of 7–8 μm. Afterward, 6 g/L Na_2_[ZnEDTA] was added to the base solution, and MAO coatings exhibited loose structures. In addition, many micropores were developed on the sample surfaces with a pore diameter of 6–7 μm. With an increase in Na_2_[ZnEDTA] concentration, the fabricated MAO coatings became more uniform with a decreased trend in pore size. According to EDS analysis, the Zn contents of Zn-6 g/L, Zn-12 g/L, Zn-18 g/L, and Zn-24 g/L were 1.22 wt.%, 1.70 wt.%, 2.62 wt.%, and 3.64 wt.%, respectively, indicating that the enhanced Na_2_[ZnEDTA] concentration could increase the Zn contents in MAO coatings. As shown in Figure 1(a3–e3), Zn-0 g/L, Zn-6 g/L, Zn-12 g/L, Zn-18 g/L, and Zn-24 g/L were 17.93 ± 0.70, 14.73 ± 0.79, 14.55 ± 0.67, 13.32 ± 1.05, and 11.74 ± 0.16 μm in thickness, suggesting that the coating became thin with the enhanced Na_2_[ZnEDTA] concentrations.

#### 3.1.2. XRD Analysis

**Figure 2:**
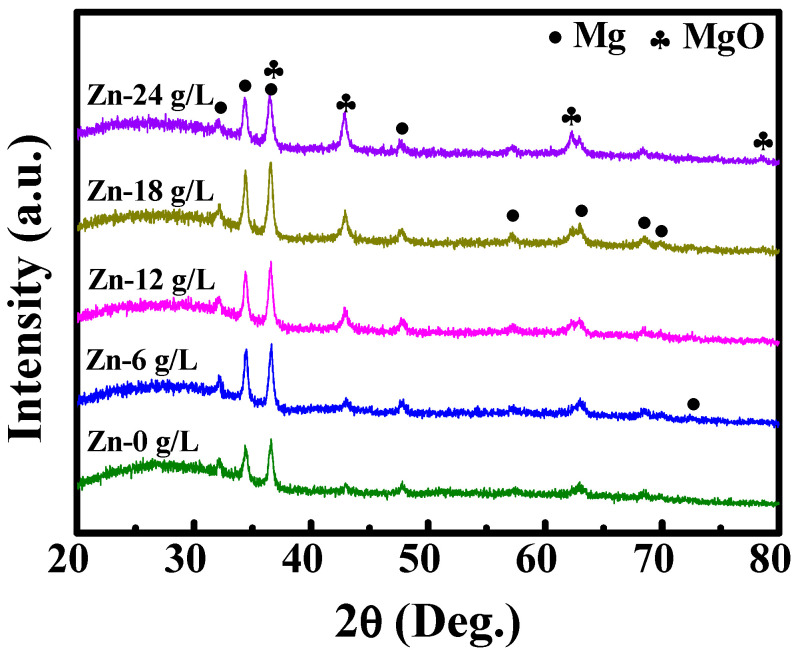
XRD patterns of MAO coatings fabricated in solutions with different concentrations of Na_2_[ZnEDTA].

XRD spectra of MAO coatings fabricated in the base solution added 0, 6, 12, 18, and 24 g/L Na_2_[ZnEDTA] and are shown in Figure 2. In the base solution without Na_2_[ZnEDTA], MAO coatings were mainly composed of Mg and MgO. After Na_2_[ZnEDTA] was added to the base solution, zinc-containing crystalline phases in MAO coatings were not detected, suggesting that these substances might be few or exist mainly in an amorphous state.

#### 3.1.3. Degradation Resistance

**Figure 3:**
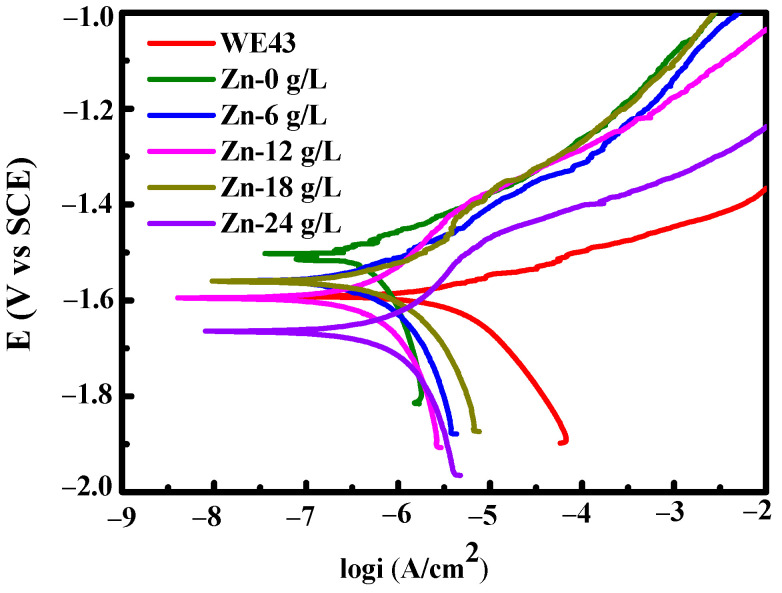
Polarization curves of MAO coatings fabricated in the solutions containing different concentrations of Na_2_[ZnEDTA].

Potentiodynamic polarization curves of untreated WE43 magnesium alloys and the fabricated MAO samples in the base solution with different Na_2_[ZnEDTA] concentrations are shown in Figure 3. The fitted electrochemical parameters using the Tafel extrapolation method are listed in Table 1. The *i*_corr_ values of the WE43 substrate and MAO samples developed in the base solution of 0, 6, 12, 18, and 24 g/L Na_2_[ZnEDTA] were 1.13 × 10^−5^, 5.75 × 10^−7^, 6.84 × 10^−7^, 1.09 × 10^−6^, 1.18 × 10^−6^, and 1.39 × 10^−6^ A·cm^−2^, respectively (Table 1). In general, a smaller *i*_corr_ exhibits better degradation resistance [35,36]. Compared with the WE43 substrate, MAO samples exhibited lower *i*_corr_ values and, therefore, better degradation resistance. According to Table 1, the MAO sample formed in the base solution with 6 g/L Na_2_[ZnEDTA] revealed the lowest *i*_corr_ value (6.84 × 10^−7^ A·cm^−2^), which was about two orders of magnitude lower than untreated WE43 alloys (1.13 × 10^−5^ A·cm^−2^). However, with an increase in Na_2_[ZnEDTA] concentration, the *i*_corr_ values of MAO samples continually enhanced, indicating that an increase in Na_2_[ZnEDTA] concentration could decrease coating degradation resistance.

### 3.2. In Vitro Cytocompatibility

#### 3.2.1. Cell Adhesion

**Figure 4:**
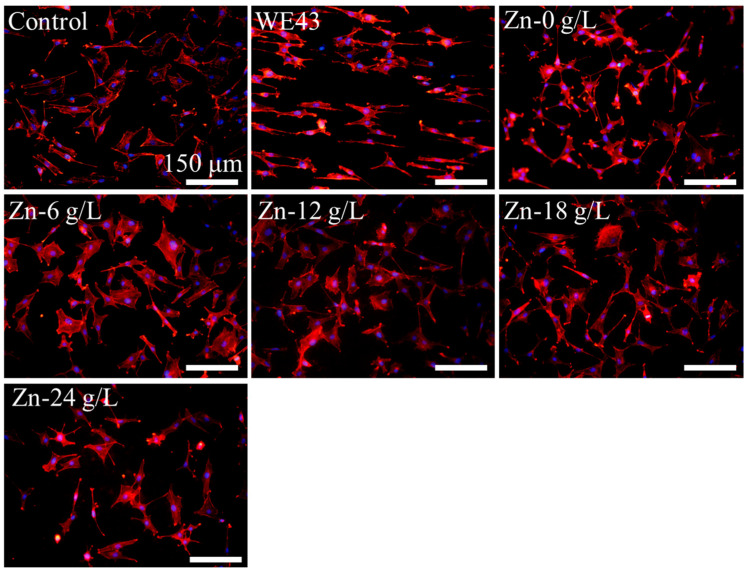
Fluorescent images of cells after culturing for 5 h on the surface of MAO coatings fabricated in the solutions containing different concentrations of Na_2_[ZnEDTA]. Nuclei and cytoskeleton are shown in blue and rede fluorescence, respectively.

Figure 4 displays the fluorescence images of cells cultured on the coatings prepared in different Na_2_[ZnEDTA] concentrations of electrolytes after 5 h of culture. It can be seen that at the initial stage of cell adhesion, most of the cells on the WE43 substrate spread along the surface scratches and revealed a slender spindle shape with fewer cell pseudopods. In comparison, the cells that adhered to MAO-treated samples presented numerous filamentous and lamellar pseudopodia, and the spreading area based on the labeled cytoskeleton (red fluorescence) was larger than the WE43 substrate, indicating that MAO-treated samples presented good cell spreading and initial cell adhesion.

#### 3.2.2. Cytotoxicity

**Figure 5:**
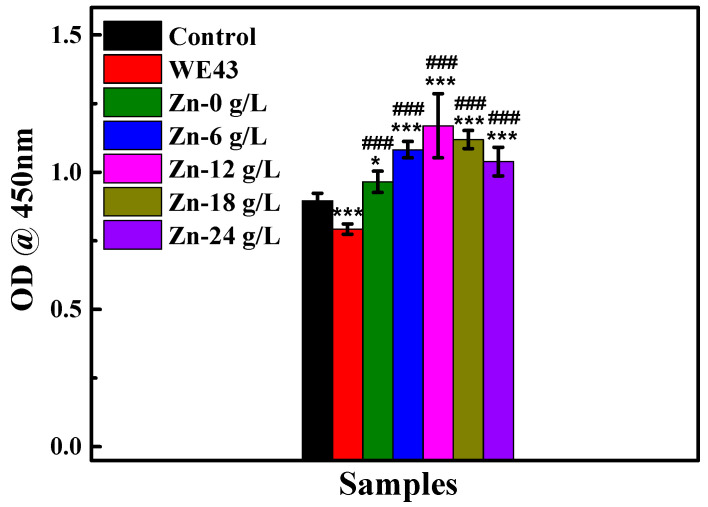
The OD value of MC3T3-E1 cells cultured with different MAO sample extracts for 3 d. *, *** *p* < 0.05, 0.001 vs. control, ### *p* < 0.001 vs. WE43.

Figure 5 shows an OD value of MC3T3-E1 cells after 3 d of cultivation in extracts of various MAO samples and the WE43 substrate. The OD value of MC3T3-E1 cells cultured in extracts of various MAO samples presents significant enhancement compared to untreated WE43 and the control, indicating that MAO samples present enhanced cell viability and good in vitro cytocompatibility.

#### 3.2.3. ALP Activity

**Figure 6:**
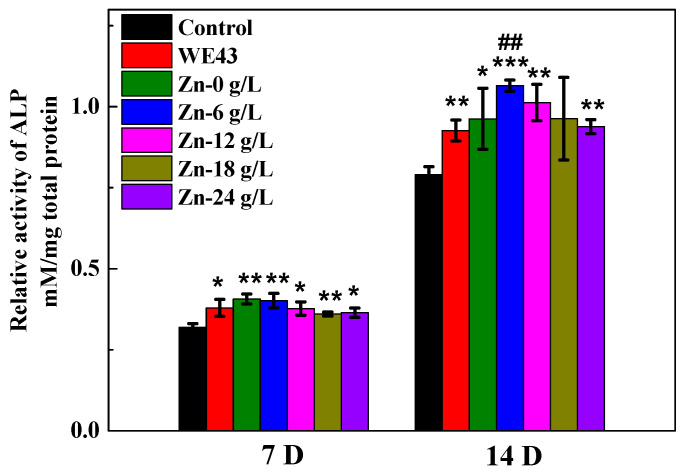
The relative activity of the ALP of MC3T3-E1 pre-osteoblasts cultured on the surface of MAO coatings fabricated in solutions containing different concentrations of Na_2_[ZnEDTA] for 7 and 14 d. *, **, *** *p* < 0.05, 0.01, 0.001 vs. control, ## *p* < 0.01 vs. WE43.

Figure 6 shows the ALP activity of MC3T3-E1 cells cultured on the MAO samples and WE43 substrate for 7 and 14 d. After 7 d and 14 d of culture, MC3T3-E1 cells cultured on untreated WE43 and MAO coatings both showed significantly higher ALP activity than the control group, but there is no significant difference between the MAO coatings and WE43 substrate, except for between Zn-6 g/L and the WE43 substrate after 14 d of culture. Overall, MAO samples revealed beneficial promotion in osseogenic differentiation; particularly, Zn-6 g/L presented the best osteoblast differentiation ability among the MAO samples.

## 4. Discussion

### 4.1. Formation of Zinc-Containing MAO Coatings

In this study, zinc-containing MAO coatings were fabricated on magnesium alloys, and the effects of Na_2_[ZnEDTA] concentration on coating characteristics, including chemical compositions, corrosion resistance, and especially in vitro cytocompatibility, were studied. Since magnesium is very active, it is difficult to develop zinc-containing coatings on magnesium alloys by one-step MAO treatment. At present, zinc-containing electrolytes, including Na_2_[ZnEDTA] [27,28] and ZnO particles [29,30], have been selected to fabricate MAO coatings. Additionally, some researchers used two-step methods to develop zinc-containing MAO coatings on magnesium alloys [37,38].

In our previous study [28], the influencing mechanism of the used electrolytes on corrosion resistance and the Zn amount of MAO coatings were revealed using an orthogonal experiment. Zinc is an amphoteric metal and in an acid solution, Zn^2+^ ions are the main existing state. However, Zn(OH)_3_^−^ or Zn(OH)_4_^2−^ ions are mainly present in an alkaline solution. In this study, the near-neutral solutions were used and, therefore, Zn^2+^ and Zn(OH)_3_^−^ or Zn(OH)_4_^2−^ ions may be present in MAO solutions. In addition, the used solutions include strong chelating agents, such as EDTA^4−^ and phytic acid, which can promote Zn^2+^ ions entering into MAO coatings [28]. With the enhanced Na_2_[ZnEDTA] concentrations, the Zn amount in MAO coatings continually increased, indicating that Zn^2+^ ions entered into MAO coatings, mainly by diffusion.

### 4.2. Property of Zinc-Containing Coatings

The biocompatibility of MAO coatings is influenced by many factors, including coating composition [31,37], corrosion resistance [37], wettability [39], and pore size [40]. Compared to the substrate, the fabricated samples by MAO treatment exhibit good cytocompatibility, which may be mainly attributed to their good degradation resistance and biocompatible chemical compositions.

For MAO-treated magnesium alloys, the cytocompatibility is closely related to its corrosion resistance. In corrosive environments, the rapid degradation of MAO coatings is accompanied by hydrogen evolution and a subsequent increase in local pH values, which usually results in cell death and tissue inflammation [37]. Therefore, MAO coatings with good degradation resistance can restrict the fast degradation of magnesium alloys and, therefore, may potentially improve their cytocompatibility. Among four zinc-containing MAO samples, the sample fabricated in the base solution added 6 g/L Na_2_[ZnEDTA] and achieved the best degradation resistance and the best osteoblast differentiation ability.

The coating composition is another important influencing factor on its cytocompatibility. The fabricated MAO coatings in this study mainly contain fluorine (F), P, and Zn. F is an essential trace element, and a proper F amount is beneficial for human health. According to our previous results, MAO coatings with low F content (3.49 at.%) achieve good cytocompatibility, while those with high F content (higher than 19.00 at.%) exhibit high cytotoxicity [19]. According to Figure 1(a2–e2), the F contents in zinc-containing coatings developed in the base solution added 0, 6, 12, 18, and 24 g/L Na_2_[ZnEDTA] and are 8.42 at.%, 3.63 at.%, 11.34 at.%, 8.40 at.%, and 7.01 at.%, illustrating that F cannot produce toxicity in bone cells. In addition to F, P is an important component in MAO coatings. As a macro element, P accounts for about 1% of human body mass. Related studies showed that pre-osteoblasts on a P-containing coating exhibited significant improvements in adhesion, proliferation, and differentiation [41]. Moreover, Zn is an essential trace element, and Zn-doped MAO coatings can increase the cytocompatibility of magnesium alloys [31]. In addition, materials with Zn ions can potentially enhance osteogenic differentiation ability [42]. It has been found that Zn can promote osteogenesis in a dose–dependent manner. Wang et al. [43] reported that the scaffold with 2 wt.% Zn was optimal for bone regeneration. In this study, among the MAO samples developed in the base solution added to 0, 6, 12, 18, and 24 g/L Na_2_[ZnEDTA], the Zn-6 g/L sample presented the lowest Zn content of 1.22 wt.% but achieved the best degradation resistance and osseogenic ability. This indicated that the introduction of Zn to MAO coatings was favorable to improving the induction ability of osseogenic differentiation. The enhancement of degradation resistance for magnesium played a more important role in the enhancement of osseogenic ability compared to Zn content in MAO coatings.

## 5. Conclusions

In the neutral solution, the influences of Na_2_[ZnEDTA] concentration on the morphology, composition, degradation resistance, and cytocompatibility of zinc-containing MAO coatings were studied. The following conclusions can be summarized:

1. Zn^2+^ ions entered into MAO coatings mainly by diffusion. XRD analysis shows that MAO coatings are mainly composed of Mg and MgO;
2. MAO treatment can improve the degradation resistance of untreated magnesium alloys. However, the enhanced Na_2_[ZnEDTA] concentration decreases the coating thickness and degradation resistance;
3. Zinc-containing MAO coatings have good cytocompatibility, and among the received WE43 substrate and zinc-containing MAO samples, the sample developed in the base solution added 6 g/L Na_2_[ZnEDTA] and achieved the best degradation resistance and osseogenic ability.

## Figures and Tables

**Table 1:** The electrochemical parameters of MAO coatings fabricated in the solutions containing different concentrations of Na_2_[ZnEDTA].

Solutions	*βa* (mV/dec)	*βc* (mV/dec)	*i*_corr_ (A·cm^−2^)	*E*_corr_ (V)
Substrate	313.21	316.26	1.13 × 10^−5^	−1.6045
Zn-0 g/L	101	475.98	5.75 × 10^−7^	−1.5028
Zn-6 g/L	135.89	285.77	6.84 × 10^−7^	−1.5603
Zn-12 g/L	229.76	412.33	1.09 × 10^−6^	−1.5952
Zn-18 g/L	221.46	311.66	1.18 × 10^−6^	−1.5631
Zn-24 g/L	232.06	397.92	1.39 × 10^−6^	−1.6655

## Data Availability

Data are contained within the article.
